# Supplementary material for: Practical intelligent diagnostic algorithm for wearable 12-lead ECG via self-supervised learning on large-scale dataset
Source: Nat Commun. 2023 Jun 23;14:3741. doi: 10.1038/s41467-023-39472-8 (PMC10290151; doi:10.1038/s41467-023-39472-8)
Supplement: Supplementary file 2 — Description of Additional Supplementary Files [file 41467_2023_39472_MOESM2_ESM.pdf]

Description of Additional Supplementary Files

Supplementary Data 1
Title: All ECG labels and its subordination of our database. These labels are two levels of catalogue, and the 60 labels that our model can currently recognize are marked in red.

Description: Our ultimate goal is to use a deep learning model to recognize and detect all these labels, but the lack of rare class samples is the main factor limiting the classification performance of the model to practical. Therefore, when the amount of samples in the secondary catalogue is insufficient, we can only merge some of the secondary labels and then recognize their parent catalogue. In the future, we will make our best efforts to collect ECGs of rare class with a small number of samples, and to expand the range of recognizable labels as much as possible so that more cardiac rhythms can be recognized by the model.
