# Supplementary material for: Practical intelligent diagnostic algorithm for wearable 12-lead ECG via self-supervised learning on large-scale dataset
Source: Nat Commun. 2023 Jun 23;14:3741. doi: 10.1038/s41467-023-39472-8 (PMC10290151; doi:10.1038/s41467-023-39472-8)
Supplement: Supplementary file 4 — Reporting Summary [file 41467_2023_39472_MOESM4_ESM.pdf]

nature portfolio

Corresponding author(s): Wei Yang, Qianjin Feng

Last updated by author(s): May 24, 2023

Reporting Summary

Nature Portfolio wishes to improve the reproducibility of the work that we publish. This form provides structure for consistency and transparency in reporting. For further information on Nature Portfolio policies, see our Editorial Policies and the Editorial Policy Checklist.

Statistics

For all statistical analyses, confirm that the following items are present in the figure legend, table legend, main text, or Methods section.

n/a	Confirmed	
☐	☒	The exact sample size (n) for each experimental group/condition, given as a discrete number and unit of measurement
☐	☒	A statement on whether measurements were taken from distinct samples or whether the same sample was measured repeatedly
☐	☒	The statistical test(s) used AND whether they are one- or two-sided<br>*Only common tests should be described solely by name; describe more complex techniques in the Methods section.*
☒	☐	A description of all covariates tested
☒	☐	A description of any assumptions or corrections, such as tests of normality and adjustment for multiple comparisons
☐	☒	A full description of the statistical parameters including central tendency (e.g. means) or other basic estimates (e.g. regression coefficient) AND variation (e.g. standard deviation) or associated estimates of uncertainty (e.g. confidence intervals)
☐	☒	For null hypothesis testing, the test statistic (e.g. F, t, r) with confidence intervals, effect sizes, degrees of freedom and P value noted<br>*Give P values as exact values whenever suitable.*
☒	☐	For Bayesian analysis, information on the choice of priors and Markov chain Monte Carlo settings
☒	☐	For hierarchical and complex designs, identification of the appropriate level for tests and full reporting of outcomes
☒	☐	Estimates of effect sizes (e.g. Cohen's d, Pearson's r), indicating how they were calculated

Our web collection on statistics for biologists contains articles on many of the points above.

Software and code

Policy information about availability of computer code

Data collection	No software was used, and the open-source code of our study is available at GitHub: https://github.com/SMU-MedicalVision/ECG-Classfication and Zenodo: https://doi.org/10.5281/zenodo.7964774.
Data analysis	All analyses were done in python 3.7.11. Python packages used are h5py (3.6.0), scipy (1.7.3), numpy (1.21.5), pandas (1.3.5), sklearn (1.0), matplotlib (3.5.1), neurokit2 (0.1.7), pytorch (1.10.1+cu102) and torchvision (0.11.2+cu102).We used all ECGs to train a Siamese network via contrastive learning, then transferred the pretrained weights to the downstream classification network. All analysis of three strategies: pre-training weights (PW), multi-scale convolution and data augmentation operations (Aug) were conducted on scipy.stats. We plotted the PRC curve with matplotlib and calculated AUC on sklearn.metrics. The architecture of network and training processes are based on pytorch.

For manuscripts utilizing custom algorithms or software that are central to the research but not yet described in published literature, software must be made available to editors and reviewers. We strongly encourage code deposition in a community repository (e.g. GitHub). See the Nature Portfolio guidelines for submitting code & software for further information.

Data

Policy information about availability of data

All manuscripts must include a data availability statement. This statement should provide the following information, where applicable:

- Accession codes, unique identifiers, or web links for publicly available datasets
- A description of any restrictions on data availability
- For clinical datasets or third party data, please ensure that the statement adheres to our policy

Source data for all experimental results are provided with this paper. In addition, for academic purposes we have made the offline test set total of 7000 ECGs publicly available at ScienceDB: https://doi.org/10.57760/sciencedb.07677.

Human research participants

Policy information about studies involving human research participants and Sex and Gender in Research.

Reporting on sex and gender	Among our large-scale ECG dataset, 67.3% ECGs were from male and the remaining 32.7% were from female.The occurrence of various ECG waveform changes and arrhythmias does not differ significantly by sex, and cardiologists do not take sex into account when making ECG diagnoses. Therefore sex was not considered in this study, nor was there any specific treatment or design for sex.
Population characteristics	Our ECGs are all collected from China and cover all provinces, autonomous regions and municipalities in China. There are three main sources of these data: home users who wear the wearable devices to collect ECGs at home and upload, patients in hospitals who use wearable devices to collect and upload, and the company and the hospital organize free ECG diagnostic events to collect ECG data. Our dataset covers ECGs from human participants aged 8 to 94 years, with an average age of 51 years. Overall, the younger participants had a healthier cardiovascular system and their ECGs were mostly normal. The prevalence of various cardiac arrhythmias gradually increased with age.
Recruitment	Informed consent was obtained from all human participants.
Ethics oversight	This study has been approved, evaluated and medically guided by the Department of Cardiology of Chinese PLA General Hospital.

Note that full information on the approval of the study protocol must also be provided in the manuscript.

Field-specific reporting

Please select the one below that is the best fit for your research. If you are not sure, read the appropriate sections before making your selection.

☒ Life sciences ☐ Behavioural & social sciences ☐ Ecological, evolutionary & environmental sciences

For a reference copy of the document with all sections, see nature.com/documents/nr-reporting-summary-flat.pdf

Life sciences study design

All studies must disclose on these points even when the disclosure is negative.

Sample size	We collected 658,486 wearable 12-lead ECGs, among which 164,538 were diagnosed by a cardiologist and reviewed by senior cardiologists, and the remaining 493,948 ECGs were without diagnosis information. Our dataset has a total of 60 classes and is multi-labeled, including normal rhythm, sinus rhythm, 8 waveform changes and 50 common arrhythmias.To validate our approach, we provide online real-time diagnostic services for 12,521 ECGs in the past two months, and these ECGs are used as online test set.In addition, we tested our approach on the China physiological signal challenge 2018 dataset (http://2018.icbeb.org/Challenge.html, CPSC2018).
Data exclusions	No dataset was excluded.
Replication	We validated our approach on offline test set, online test set and CPSC2018.
Randomization	The offline test set comprised 7,000 randomly selected annotated ECGs according to identity document, and the rest of the ECGs comprised the training set. All 12,521 ECGs from the last two months were used as the online test set.The open source ECGs of CPSC2018 on its website are all in training set, and the test set is invisible.
Blinding	In the online test, the cardiologist was blinded to the AI's diagnosis, but could compare the human expert's diagnosis with the AI's diagnosis after the diagnosis. Blinding in the remaining experiments was not relevant to our study.

Reporting for specific materials, systems and methods

We require information from authors about some types of materials, experimental systems and methods used in many studies. Here, indicate whether each material, system or method listed is relevant to your study. If you are not sure if a list item applies to your research, read the appropriate section before selecting a response.

Materials & experimental systems

n/a	Involved in the study	
☒	☐	Antibodies
☒	☐	Eukaryotic cell lines
☒	☐	Palaeontology and archaeology
☒	☐	Animals and other organisms
☒	☐	Clinical data
☒	☐	Dual use research of concern

Methods

n/a	Involved in the study	
☒	☐	ChIP-seq
☒	☐	Flow cytometry
☒	☐	MRI-based neuroimaging
